# Probing the out-of-equilibrium dynamics of driven colloids by X-ray photon correlation spectroscopy

**DOI:** 10.1107/S1600576725001244

**Published:** 2025-03-07

**Authors:** Theyencheri Narayanan, William Chèvremont, Thomas Zinn

**Affiliations:** a ESRF – The European Synchrotron, 38043Grenoble, France; NSRRC, Taiwan

**Keywords:** X-ray photon correlation spectroscopy, XPCS, ultra-small-angle X-ray scattering, USAXS, colloids, out-of-equilibrium dynamics, non-equilibrium fluctuations

## Abstract

The high brilliance of fourth-generation synchrotron sources coupled with advanced X-ray detectors enables a wide range of dynamic studies of colloids and other soft-matter systems. In particular, the higher fraction of coherent flux provided by these new sources is a major boost for X-ray photon correlation spectroscopy (XPCS). As a result, not only can equilibrium dynamics be accessed but also relatively fast out-of-equilibrium processes can be investigated by XPCS. This article briefly recalls the statistical properties of coherent scattering and then demonstrates a case study of non-equilibrium fluctuations in a driven colloidal system. A simple example is the resuspension of colloids by vigorous shaking, where the inhomogeneous flow generates local variations in number density of particles leading to strong velocity fluctuations. The Brownian motion of the particles homogenizes the suspension with time and the system gradually returns to pure diffusive dynamics. On the other hand, in a uniformly sheared suspension of particles, such concentration gradients do not form and upon cessation of shear the return to Brownian dynamics is rapid. These transient non-equilibrium effects can inadvertently influence micrometre-range particle size measurement by means of dynamic scattering methods.

## Abstract 2

This article presents an application of X-ray photon correlation spectroscopy (XPCS) to probe the out-of-equilibrium dynamics in a driven colloidal system. XPCS enables the investigation of direction-dependent non-equilibrium dynamics and the gradual return of the suspension to equilibrium Brownian behaviour. The influence of such transient non-equilibrium dynamics needs to be taken into consideration when dynamic scattering methods are used for micrometre-range particle measurement.

## 1. Introduction

Fourth-generation synchrotron sources based on multi-bend achromat storage-ring lattices have come into operation in recent years (Eriksson *et al.*, 2014 ▸; Shin, 2021 ▸). These X-ray sources together with advanced photon-counting pixel array detectors (Fröjdh *et al.*, 2024 ▸) are very attractive for performing scattering experiments (Narayanan *et al.*, 2023 ▸). Compared with the previous third-generation synchrotrons, these new sources have increased the brilliance and degree of transverse coherence of X-ray beams by more than an order of magnitude (Raimondi *et al.*, 2021 ▸; Shin, 2021 ▸). Both small-angle X-ray scattering (SAXS) and X-ray photon correlation spectroscopy (XPCS) methods benefit from the enhanced brightness and coherence of the X-ray beam. Usually, SAXS experiments are performed using a larger beam consisting of multiple coherence volumes; the smaller beam cross section and lower divergence in the horizontal direction have improved the angular resolution (Narayanan *et al.*, 2022 ▸). The time resolution offered by the current advanced detectors enables probing of kinetic processes in the millisecond range and below by time-resolved SAXS and allied methods (Narayanan, 2024 ▸). Ideally, XPCS requires single or a few coherent scattering volumes (Sutton, 2008 ▸) and therefore the benefit of the new sources is even more significant (Lehmkühler *et al.*, 2021 ▸; Narayanan *et al.*, 2023 ▸). In particular, the higher degree of coherence together with the high frame rate of pixel array detectors enables multispeckle XPCS measurements in the sub-millisecond time range (Zhang *et al.*, 2018 ▸; Zinn *et al.*, 2018 ▸). Furthermore, XPCS down to the sub-microsecond range can be performed at X-ray free-electron laser (XFEL) facilities (Lehmkühler *et al.*, 2021 ▸; Dallari *et al.*, 2021 ▸).

In this article a particular type of out-of-equilibrium dynamics in a driven colloidal suspension is investigated. The suspension is vigorously shaken and then left to equilibrate until Brownian dynamics are restored. During the intervening time, strong velocity fluctuations (δ*v*) akin to a sedimenting suspension are observed. In a sedimenting colloidal suspension, the hydrodynamic back flow caused by neighbouring particles reduces the sedimentation velocity (*v*) from the Stokes velocity (*v*_S_) (Batchelor, 1972 ▸). Fluctuations in the local particle number density (*N*) lead to variance in the sedimentation speed that scales with the system size (Caflisch & Luke, 1985 ▸), 〈δ*v*^2^〉 ∼ *L*, with *L* the smallest dimension of the container. Such a diverging variance in sedimentation speed has not been observed experimentally and remains a puzzle (Ramaswamy, 2001 ▸; Guazzelli & Hinch, 2011 ▸; Segrè, 2016 ▸). However, in the present case the colloidal particles are sub-micrometre sized with Péclet number Pe (representing the relative importance of advection over diffusion) ∼0.1. That means Brownian motion dominates over gravitationally induced settling and the macroscopic sedimentation time is of the order of many hours. Nevertheless, thermally induced or Brownian velocity fluctuations can be significant even at low values of Pe and moderate volume fraction ϕ of particles (Padding & Louis, 2008 ▸).

During the shaking process in a capillary, the suspension is subjected to a high shear that is non-uniform and the long-range hydrodynamic fluctuations thereby induced decay with time. In particular, the spatial and temporal correlations of the hydrodynamic δ*v* are known to exhibit an algebraic long tail (Padding & Louis, 2008 ▸). Therefore, the measured intensity–intensity autocorrelation functions *g*_2_(*q*, *t*) can become affected by this slowly decaying hydrodynamic δ*v*. The thermally induced δ*v* also contributes to *g*_2_(*q*, *t*) but its magnitude is less significant than self-diffusion (Padding & Louis, 2008 ▸). The hydrodynamic part of δ*v* is anisotropic during sedimentation (Segrè, 2016 ▸; Padding & Louis, 2008 ▸; Möller & Narayanan, 2017 ▸) and a similar anisotropy may be present in shaken samples. As a result, multispeckle XPCS and direction-dependent analysis are critical for this study. The XPCS and SAXS experiments presented here were performed in the ultra-small-angle (UA) configuration by spatially selecting the coherent part of the X-ray beam (Chèvremont *et al.*, 2024 ▸).

## 2. Experimental methods

### 2.1. Materials

The main samples used in this study consisted of charge-stabilized silica spheres with a mean radius *R*_S_ ≃ 300 nm and standard deviation σ_*R*_ ≃ 5.6 nm dispersed in MilliQ water. The volume fraction (ϕ) of the suspensions varied from 3 × 10^−3^ to 1.5 × 10^−2^. A second set of samples with *R*_S_ ≃ 126 nm and σ_*R*_ ≃ 6.2 nm were also investigated. For the characterization of intensity statistics, a dried film of polystyrene spheres (*R*_S_ ≃ 1.015 µm and σ_*R*_ ≃ 7.0 nm) was used. For all three sets of particles, *R*_S_, σ_*R*_ and ϕ were determined from ultra-small-angle X-ray scattering (USAXS) analysis (Narayanan *et al.*, 2022 ▸).

For the shaking experiment, the suspensions were contained in quartz capillaries with internal diameter ∼1 mm and wall thickness ∼10 µm. This capillary size was chosen as a compromise for fast sample displacement and multiple scattering (Semeraro *et al.*, 2018*b* ▸). For shear experiments, the colloidal suspension was contained in a coaxial capillary shear cell coupled to a rheometer (RS6000, Thermo Scientific) (Narayanan *et al.*, 2020 ▸).

### 2.2. X-ray scattering

UA-XPCS and USAXS experiments were performed on beamline ID02 (TRUSAXS) at the ESRF, Grenoble, France (Narayanan *et al.*, 2022 ▸). The measurements covered a scattering vector range of 0.002 ≤ *q* ≤ 0.1 nm^−1^ using a sample-to-detector distance *d*_SD_ of 31 m. Here, *q* is the magnitude of the scattering vector given by *q* = (4π/λ)sin(θ/2), with θ the scattering angle and λ the X-ray wavelength ≃ 1 Å. The 2D scattering patterns were acquired by an EIGER 500K (Paul Scherrer Institute) photon-counting pixel array detector with a maximum frame rate of 23000 s^−1^ (Chèvremont *et al.*, 2024 ▸). The coherent flux at the sample position was of the order of 10^12^ photons s^−1^. For recording the static speckle patterns, a high-resolution fibre-optically coupled charge-coupled device detector (FReLoN: fast readout low noise) (Narayanan *et al.*, 2022 ▸) was also used.

A coherent beam was obtained by closing the primary slits (at 27 m from the source) to 0.15 mm × 0.15 mm and two secondary slits (at 49 and 62 m from the source) to 0.04 mm vertically and 0.015 mm horizontally. The resulting beam was roughly symmetrical with FWHM ≃ 25 µm at the sample position. The degree of coherence was varied by enlarging the gaps of the two secondary slits. From the measured 2D speckle patterns, pixel-by-pixel intensity–intensity autocorrelations were calculated using the *Dynamix* package (Paleo *et al.*, 2021 ▸). The normalized ensemble-averaged two-time correlation function (TTCF) is obtained by averaging these quantities over the desired azimuthal range for a given *q* (Chèvremont *et al.*, 2024 ▸). The time averaging of the TTCF yielded the time- and ensemble-averaged intensity–intensity autocorrelation function, represented by *g*_2_(*q*, *t*) with *t* the lag time. For USAXS, the measured 2D scattering patterns were normalized and azimuthally averaged to obtain the 1D scattering profiles denoted by *I*(*q*) (Narayanan *et al.*, 2022 ▸). Further treatment and background subtraction were performed using the *SAXSutilities* software (Sztucki, 2021 ▸).

## 3. Results and discussion

### 3.1. Characterization of coherence

This section aims to provide a comparison of the degree of coherence between the instrumental configurations used for SAXS/USAXS and XPCS at a fourth-generation synchrotron X-ray source. A distinct feature of coherent scattering is the speckles in the intensity pattern, which are generated by the interference of scattered waves from the randomly distributed structural units in the medium. As a result, speckle visibility from a disordered sample is an elegant way of characterizing the coherence of the beam. The speckle visibility can be quantified in terms of the intensity statistics (Goodman, 1985 ▸). With a fully coherent beam, the scattered electric field is a Gaussian random variable, for large values of which the probability distribution tends to be normal or Gaussian following the central limit theorem. As a result, the intensity probability distribution *P*(*I*) takes the well known negative exponential form *P*(*I*) = 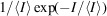, with 〈*I*〉 the mean value. In the case of a partially coherent beam, *P*(*I*) is better described by a Gamma distribution of the following form (Goodman, 1985 ▸; Abernathy *et al.*, 1998 ▸):
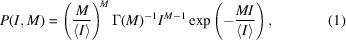
where Γ(*M*) is the Gamma function, *M* ≥ 1 is the number of modes or degrees of freedom of the distribution, and the speckle contrast β_*M*_ = 1/*M*. For *M* = 1, equation (1) reduces to the negative exponential form. Partial coherence is signified by *M* > 1 and it represents the number of coherent patches within the scattering volume. *M* → ∞ corresponds to incoherent scattering and *P*(*I*) approaches a Gaussian distribution or *I* becomes a random variable. In the literature, the conventional SAXS measured by a partially coherent beam is sometimes referred to as incoherent scattering (Grübel & Zontone, 2004 ▸; van der Veen & Pfeiffer, 2004 ▸), which is not exactly correct. Incoherent scattering usually originates from an inelastic process (Agarwal, 2013 ▸). The contribution of incoherent scattering to the measured intensity in the small-angle region is relatively low (away from an atomic absorption edge), well below 1% (Pavlinsky, 2021 ▸).

**Figure 1:**
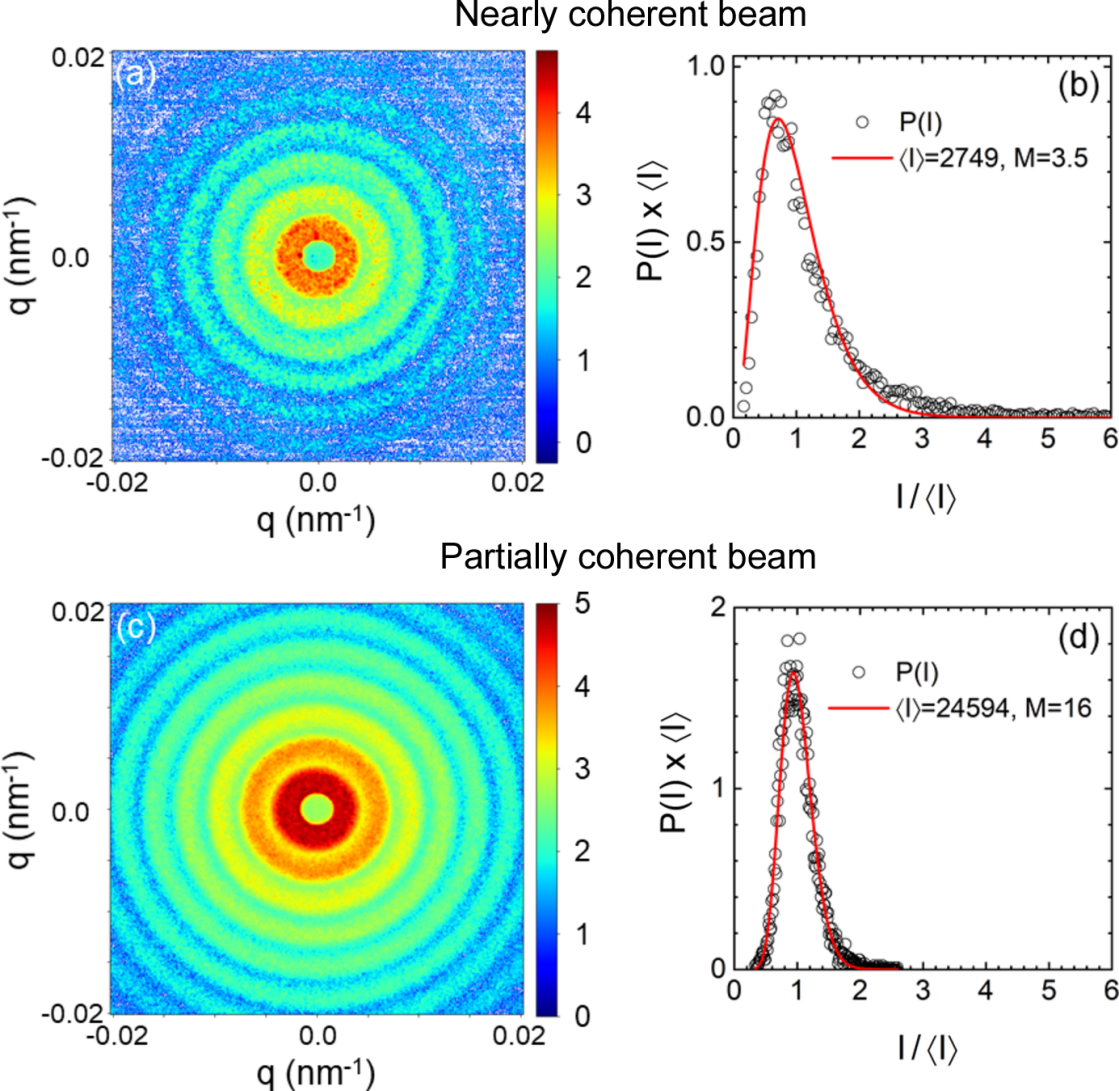
Static speckle patterns from dried polystyrene particles (*R*_S_ ≃ 1.015 µm and σ_*R*_ ≃ 7 nm) measured with two different collimation settings. The intensity statistics *P*(*I*) were analysed over the range 2.6 × 10^−3^ ≤ *q* ≤ 3.6 × 10^−3^ nm^−1^ which covers the structure peak. (*a*) Slits closed to 40 µm vertically and 20 µm horizontally. (*b*) Corresponding analysis of *P*(*I*) using equation (1). For display purposes (unit area), *P*(*I*) has been multiplied by the mean intensity 〈*I*〉. (*c*) The speckle pattern of the same sample recorded with a larger beam, slits opened to 100 µm × 100 µm, illustrating the reduction in visibility. (*d*) Corresponding *P*(*I*) and analysis using equation (1).

Fig. 1 ▸ displays static speckle patterns measured from a film of dried polystyrene particles (*R*_S_ ≃ 1.015 µm and σ_*R*_ ≃ 7 nm) with two different collimation settings at *d*_SD_ of 31 m using the high-resolution FReLoN detector (Narayanan *et al.*, 2022 ▸). In the upper panel, a nearly coherent beam was obtained by closing the last collimation slits to 40 µm (vertical) and 20 µm (horizontal), resulting in a beam size of 25 µm at the sample position. *P*(*I*) in Fig. 1 ▸(*b*) can be fitted to equation (1) with *M* = 3.5. To obtain a better fit of the peak, a larger value of *M* is needed (4.5), while the tail of the distribution requires a smaller *M* (2.5). Note that double or multiple scattering events may slightly distort the functional form of *P*(*I*) (Semeraro *et al.*, 2018*b* ▸). With the larger beam used for USAXS, the speckle visibility is significantly diminished and the corresponding *P*(*I*) in Fig. 1 ▸(*d*) is well described by equation (1) with *M* = 16. With a further reduction in speckle visibility, the value of *M* increases, and therefore *M* is a good parameter to quantify the degree of coherence. Additional data from a powder sample are shown in the supporting information (Fig. S1).

However, speckle visibility depends not only on the degree of coherence but also on the resolution of the detection scheme. The speckle size *l*_s_ scales as *l*_*s*_ ∼ λ*d*_SD_/σ_B_, with σ_B_ the size of the beam (Lehmkühler *et al.*, 2021 ▸). The speckle visibility will be very poor if the detector does not have sufficient spatial and temporal resolution or if the beam size is large. In conventional SAXS, the beam sizes used are relatively large compared with the typical transverse coherence length (ξ_T_) and therefore the speckle size becomes much smaller than the detector pixel size. This does not imply that the scattering is completely incoherent, and the phase relationship should be maintained at the largest size scale probed by the SAXS experiment (Glatter, 2002 ▸). The required level of coherence is provided by the beam collimation as ξ_T_ ≃ λ/(2ΔΘ), with ΔΘ the angular source size (Grübel & Zontone, 2004 ▸).

The coherence length determines the ultimate resolution that can be obtained (Petukhov *et al.*, 2015 ▸). The SAXS intensity from an isolated object (form factor) is the coherent sum of the scattering amplitudes from the different regions of that object. In the dilute case, the scattered intensities from different objects or particles in a suspension and the solvent molecules are incoherently summed. This is because the relative positions of other particles and solvent molecules are randomized within the typical acquisition time and the phase factor is averaged out to zero (Pusey, 2002 ▸). That is why the solvent background scattering can be subtracted out without having to deal with the cross term. In a concentrated system, the structure factor accounts for the correlation between different particles and the resulting alteration of the scattered intensity.

**Figure 2:**
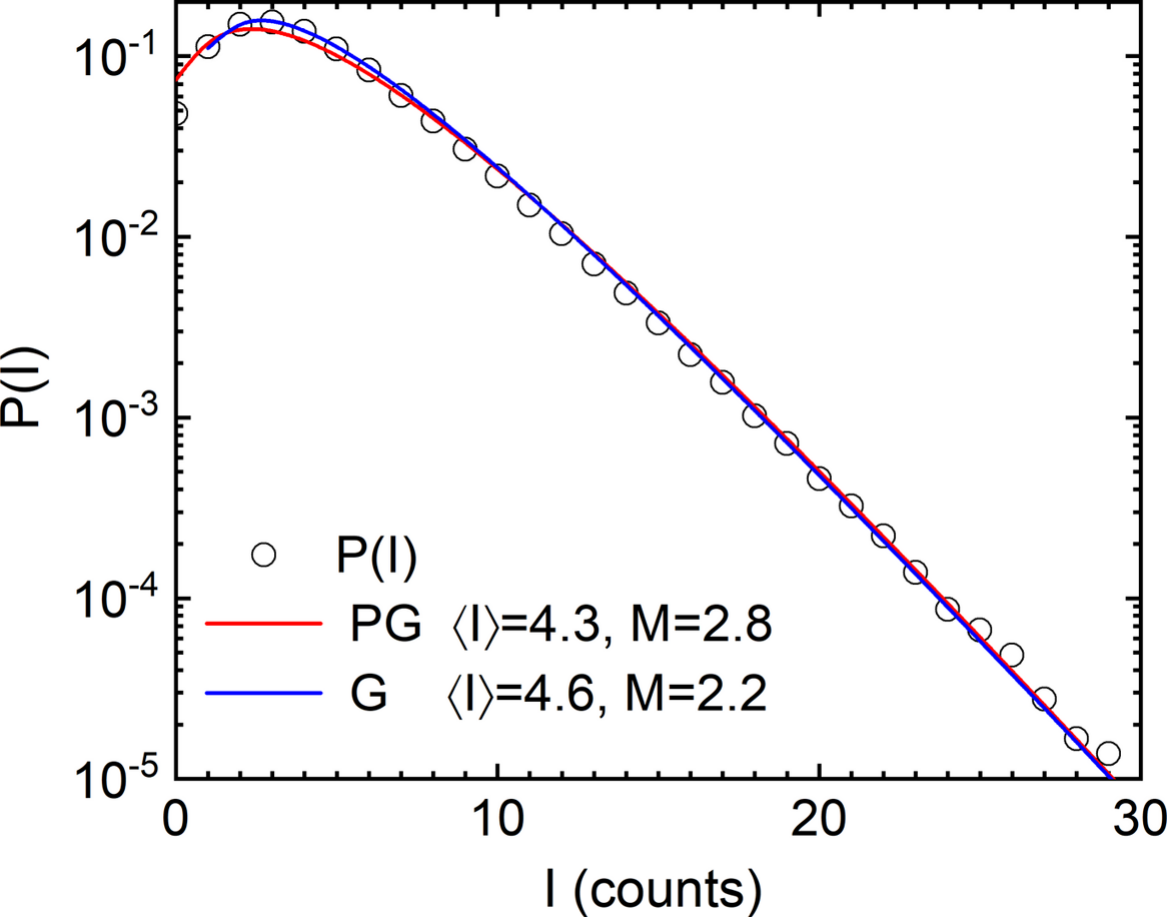
Intensity statistics *P*(*I*) for a dilute suspension of silica colloidal particles (*R*_S_ ≃ 300 nm and σ_*R*_ ≃ 5.4 nm) over the range 2.0 × 10^−3^ ≤ *q* ≤ 3.2 × 10^−3^ nm^−1^. The analysis was done in terms of equations (1) and (2) (labelled G and PG, respectively). The resulting *M* and 〈*I*〉 values are indicated in the legend.

For a more optimized setup with collimation slits further closed to 30 µm (vertical) and 15 µm (horizontal), the speckle visibility further improved and therefore *M* decreased. Fig. 2 ▸ presents the corresponding *P*(*I*) recorded with a dilute aqueous suspension of silica colloidal particles (*R*_S_ ≃ 300 nm and σ_*R*_ ≃ 5.4 nm) with an acquisition time (50 µs) much shorter than the diffusion time. In this case, *M* ≃ 2.2 and β_*M*_ ≃ 0.45. For lower intensities, *P*(*I*) is better described by the Poisson–Gamma or negative binomial distribution *P*_*M*_(*I*) (Lehmkühler *et al.*, 2021 ▸; Chèvremont *et al.*, 2024 ▸) that has the form
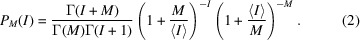
The corresponding analysis yielded a larger value of *M* ≃ 2.8. In other words, equation (1) needs to be convoluted by the Poisson statistics to estimate *M* correctly at low intensity values (Goodman, 1985 ▸).

### 3.2. Non-equilibrium velocity fluctuations

The vast majority of studies of velocity fluctuations are focused on suspensions involving non-Brownian particles (Segre *et al.*, 1997 ▸; Guazzelli & Hinch, 2011 ▸; Segrè, 2016 ▸). However, similar velocity fluctuations have been found with Brownian particles using different experimental methods (Möller & Narayanan, 2017 ▸; Hirano & Norisuye, 2024 ▸) and mesoscopic computer simulations (Padding & Louis, 2008 ▸). Incomplete mixing creates a non-random distribution of particles that generates these transient fluctuations (Padding & Louis, 2008 ▸; Guazzelli & Hinch, 2011 ▸). In the following experiment, a Brownian colloidal suspension (Pe ≃ 0.08) contained in a capillary (diameter ∼1 mm) was shaken rapidly upside down and back, such that a column of sample (20 mm) near the top was displaced at about 40 mm s^−1^ to the very bottom. During the shaking process, the induced flow can be considered as Poiseuille type having a parabolic velocity profile. This flow field generates a maximum shear rate (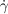) at the capillary wall and a minimum at the centre. This variation in 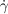 can induce long-range concentration inhomogeneities if the particles do not strictly follow the fluid due to the difference in their densities.

**Figure 3:**
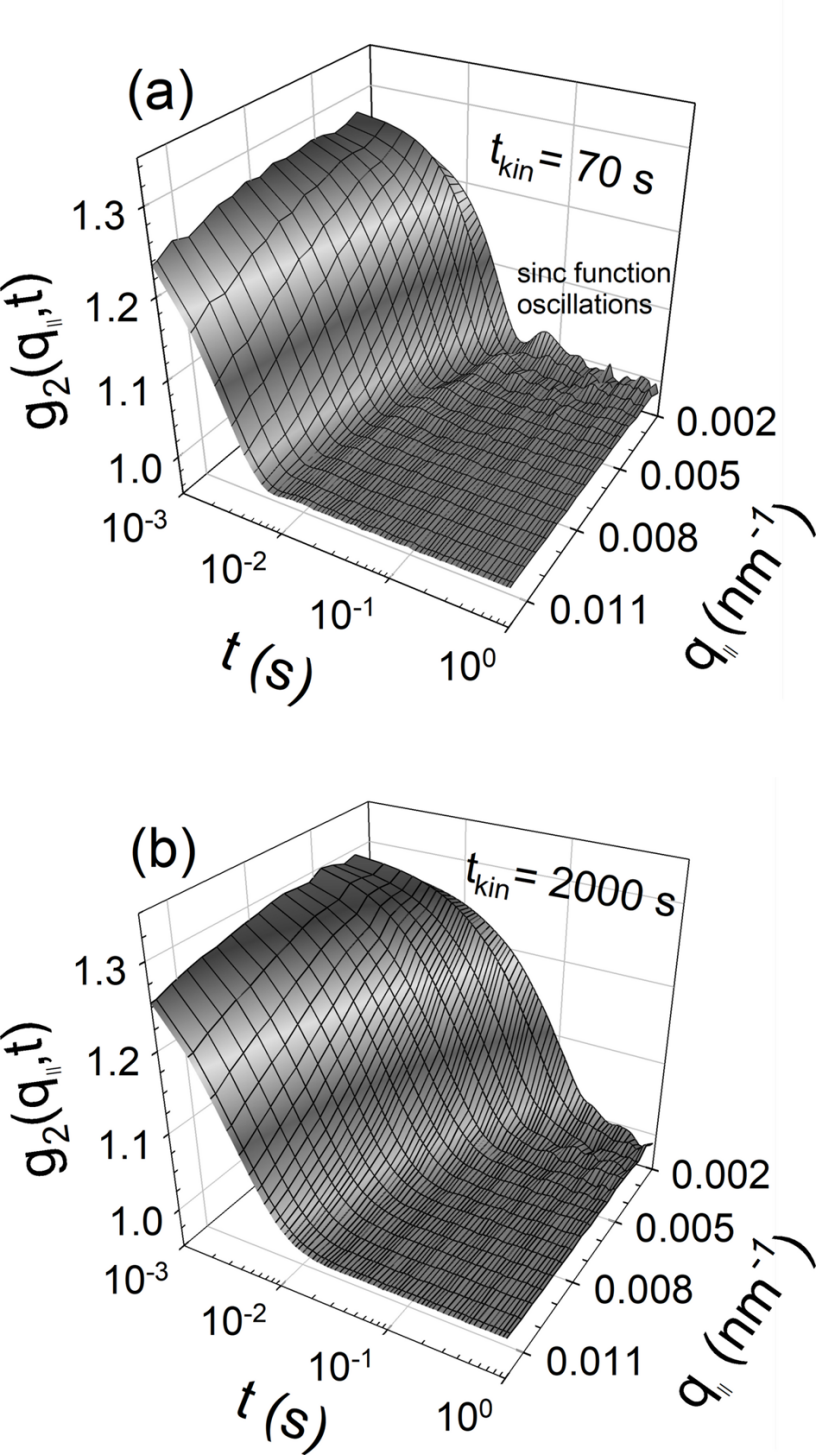
The time evolution of *g*_2_(*q*, *t*) along *q*_∥_ for a suspension of silica particles (*R*_S_ ≃ 300 nm and σ_*R*_ ≃ 5.4 nm) with ϕ ≃ 0.014. (*a*) At 70 s after shaking, illustrating the signature of velocity fluctuations (oscillations after the initial fast decay). (*b*) At about 2000 ▸ s when the suspension had returned to Brownian dynamics as characterized by a slower exponential decay. Note the differences in the *q* dependence and decay time between the two plots.

XPCS measurements were started from 70 s after shaking – the time required to interlock the experimental station. The time elapsed after shaking is represented by the kinetic time *t*_kin_. In order to avoid any beam-induced effects, the incident intensity was attenuated to 10^11^ photons s^−1^ and the number of frames was limited to 2000 ▸. Fig. 3 ▸ presents the typical time evolution of sector-averaged (±10°) *g*_2_(*q*, *t*) along the vertical direction (*q*_∥_) or axis of the capillary following the vigorous shaking (three times). The suspension consisted of silica particles (*R*_S_ ≃ 300 nm and σ_*R*_ ≃ 5.4 nm) with ϕ ≃ 0.014. Clearly, a strong deviation from diffusive dynamics can be observed 70 s after the shaking, as depicted in Fig. 3 ▸(*a*). *g*_2_(*q*_∥_, *t*) decays much faster than expected for diffusive dynamics and exhibits periodic modulations after the initial decay. These oscillations are a signature of directed motion of particles in a deterministic flow (Möller & Narayanan, 2017 ▸; Zinn *et al.*, 2020 ▸). Similar features are also observed in the horizontal direction (*q*_⊥_) along the diameter of the capillary but with a rate two to three times slower. With time these oscillations smear out and the dynamics gradually return to Brownian behaviour, characterized by an exponential decay of *g*_2_(*q*_∥_, *t*) as shown in Fig. 3 ▸(*b*). Additional plots of *g*_2_(*q*_∥_, *t*) and *g*_2_(*q*_⊥_, *t*) at an intermediate *t*_kin_ are presented in Fig. S2.

**Figure 4:**
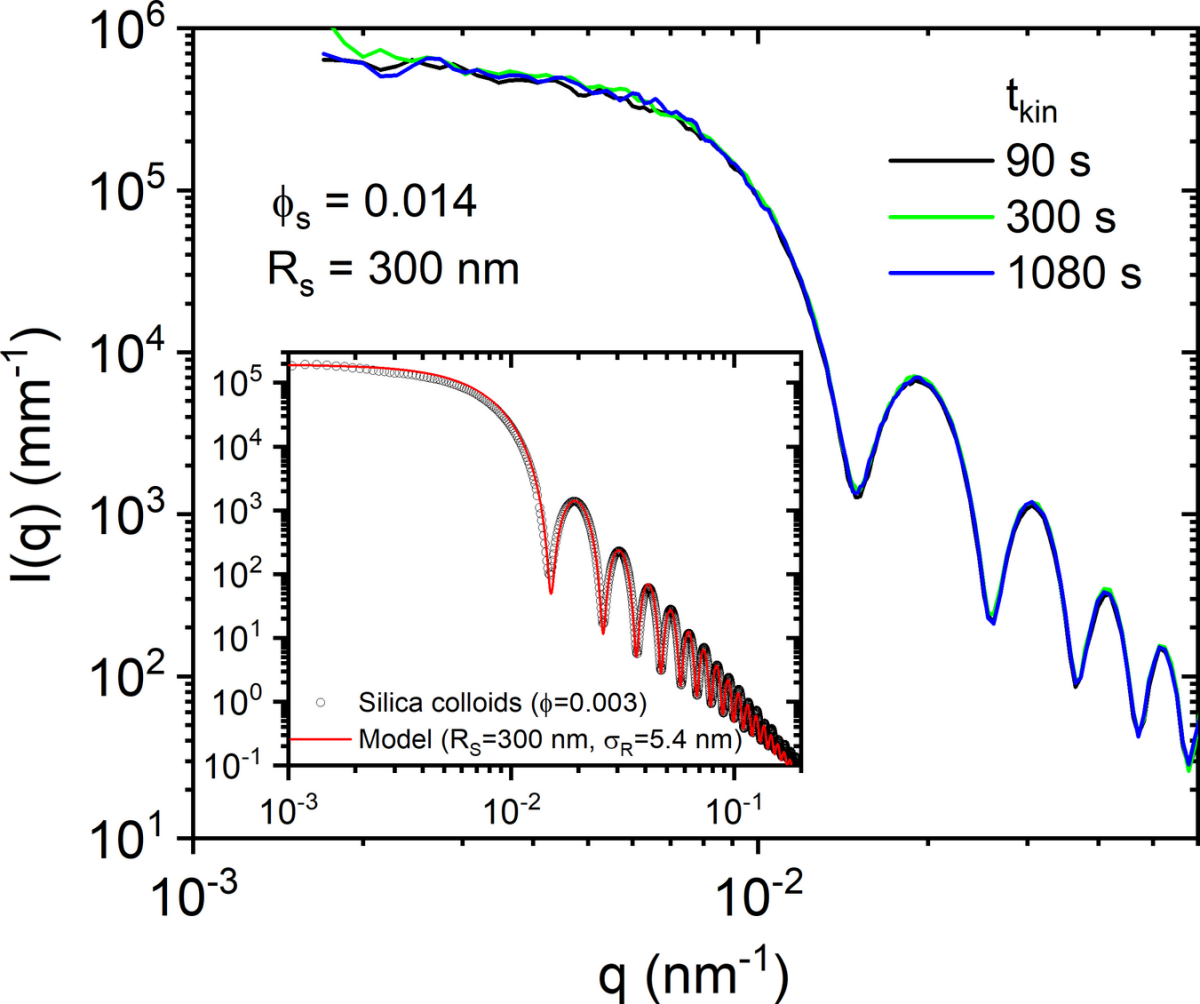
USAXS profiles of the silica colloidal suspension (*R*_S_ ≃ 300 nm and ϕ ≃ 0.014) at different times after shaking. The inset displays the scattering form factor of the particles measured over an extended *q* range using a dilute sample (ϕ ≃ 0.003). The fit corresponds to the polydisperse sphere form factor (*R*_S_ ≃ 300 nm and σ_*R*_ ≃ 5.4 nm).

The above observation is a purely dynamic effect and the static USAXS profiles do not show any significant anisotropy or variation with time. Fig. 4 ▸ displays the normalized USAXS profiles at different times after the shaking. As these particles are charge stabilized, there is a weak structure factor even at ϕ ≃ 0.014. The inset presents the form factor of these particles and the corresponding modelling in terms of the polydisperse sphere form factor with *R*_S_ ≃ 300 nm and σ_*R*_ ≃ 5.4 nm. As the profiles superimpose perfectly at different times, sedimentation effects are not important in this case. The first minima of the profiles are slightly smeared due to non-negligible multiple scattering with a sample thickness of ∼1 mm (Semeraro *et al.*, 2018*b* ▸); this is not a resolution effect as it does not happen with a dilute sample ϕ ≃ 0.003. The wiggles in the low-*q* region are due to insufficient time averaging (within an acquisition time of 0.1 s), as the speckles fluctuate more slowly at lower *q*.

The measured *g*_2_(*q*, *t*) can be related to the intermediate scattering function *g*_1_(*q*, *t*) via the Siegert relation, 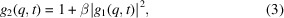
where β ≃ β_*M*_ is determined by the coherence properties of the X-ray beam and the angular resolution of the setup (Sutton, 2008 ▸). For dilute Brownian particles, *g*_1_(*q*, *t*) = 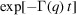 and the relaxation rate Γ(*q*) = *D*_0_*q*^2^. Here, *D*_0_ is the diffusion coefficient given by the Stokes–Einstein relation, *D*_0_ = *k*_B_*T*/(6πη*R*_H_), with *k*_B_, *T*, η and *R*_H_ being the Boltzmann constant, absolute temperature, solvent viscosity and mean hydrodynamic radius of particles, respectively.

In a driven system, advection dominates over diffusion. In this case, *g*_1_(*q*, *t*) in a given direction can be factorized in the following form, at least for relatively dilute suspensions (Busch *et al.*, 2008 ▸; Burghardt *et al.*, 2012 ▸): 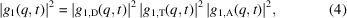
where the first term represents diffusive motions, the second term takes into account the decorrelation due to the transit of particles across the beam determined by their mean velocity *v*, and the last term denotes the advective part constituted by the differences in Doppler shift of all particle pairs in the scattering volume. This last term is determined by the average velocity difference between all particle pairs δ*v*, and the exact functional form of *g*_1,*A*_(*q*, *t*) depends on the probability distribution of *v* (Zinn *et al.*, 2020 ▸).

The oscillatory feature in Fig. 3 ▸(*a*) indicates a constant δ*v* with only a fraction α of particles at velocities deviating from *v* (Möller & Narayanan, 2017 ▸). In this case, *g*_2_(*q*, *t*) along a given direction can be expressed as
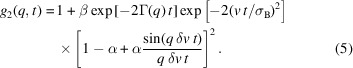
When α = 1, equation (5) reduces to that used to describe a constant velocity difference in a Couette flow (Burghardt *et al.*, 2012 ▸). α < 1 is indicated by non-zero values of minima in *g*_1_(*q*, *t*).

**Figure 5:**
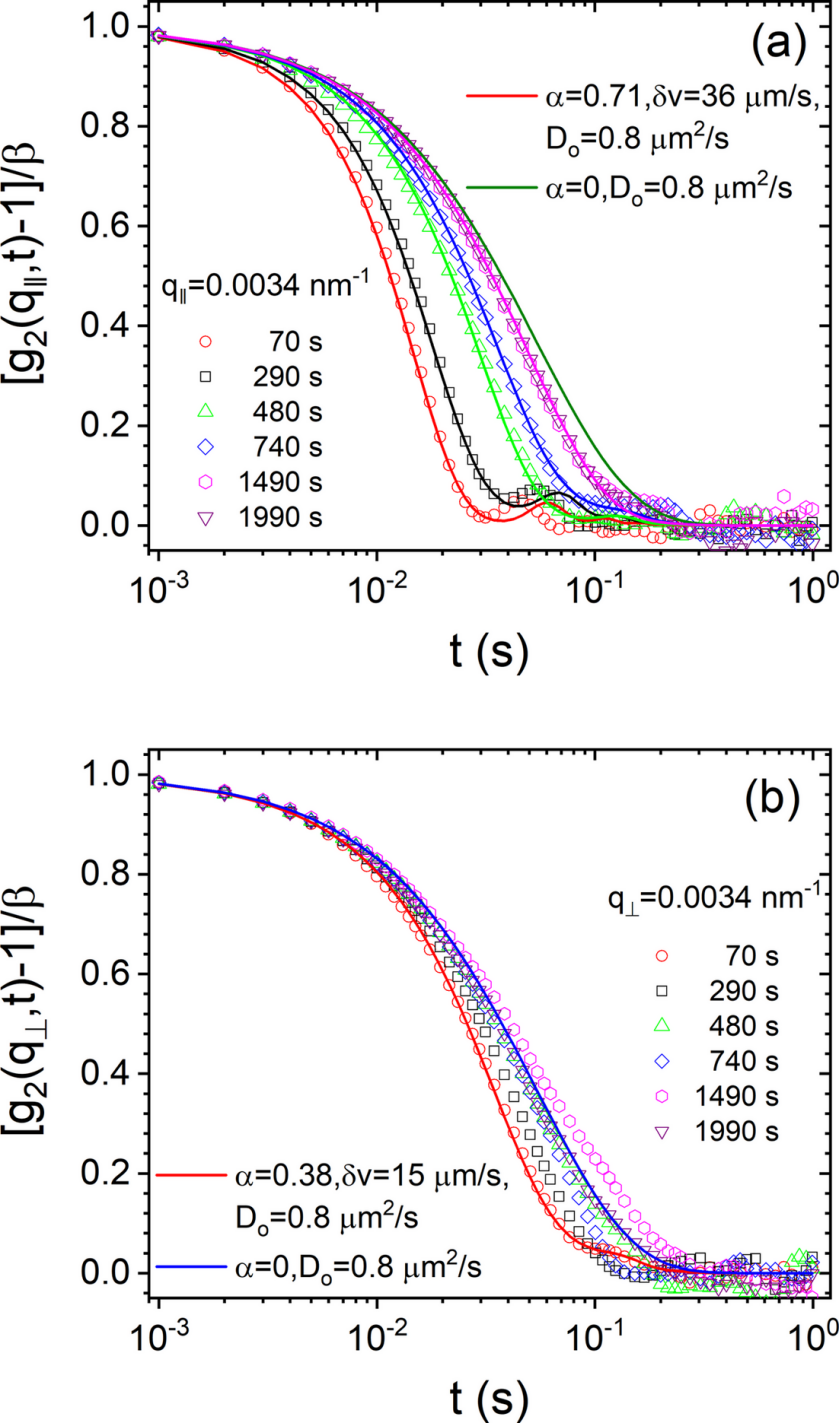
Typical time evolution of *g*_2_(*q*, *t*) along (*a*) *q*_∥_ and (*b*) *q*_⊥_ for a suspension of silica particles (*R*_S_ ≃ 300 nm and σ_*R*_ ≃ 5.4 nm) with ϕ ≃ 0.014 at fixed *q* of 3.4 × 10^−3^ nm^−1^. The continuous lines are fits to equation (5) with the values of the parameters indicated in the legends.

In the simultaneous fitting of *g*_2_(*q*, *t*) functions over 0.002 ≤ *q* ≤ 0.02 nm^−1^, both α and δ*v* were constrained to the same values at any given *t*_kin_. While δ*v* decayed systematically with *t*_kin_, α fluctuated between 0.2 and 1. Fig. 5 ▸ displays representative fits for the direction-dependent analysis of *g*_2_(*q*, *t*). The position and depth of the minima determined δ*v* and α, respectively. This coexistence of advective and diffusive motions is somewhat reminiscent of fractional Brownian motion (Nolte, 2024 ▸). Finally, the *g*_2_(*q*, *t*) functions became identical to an exponential function and at this point δ*v* and α were not determinable.

**Figure 6:**
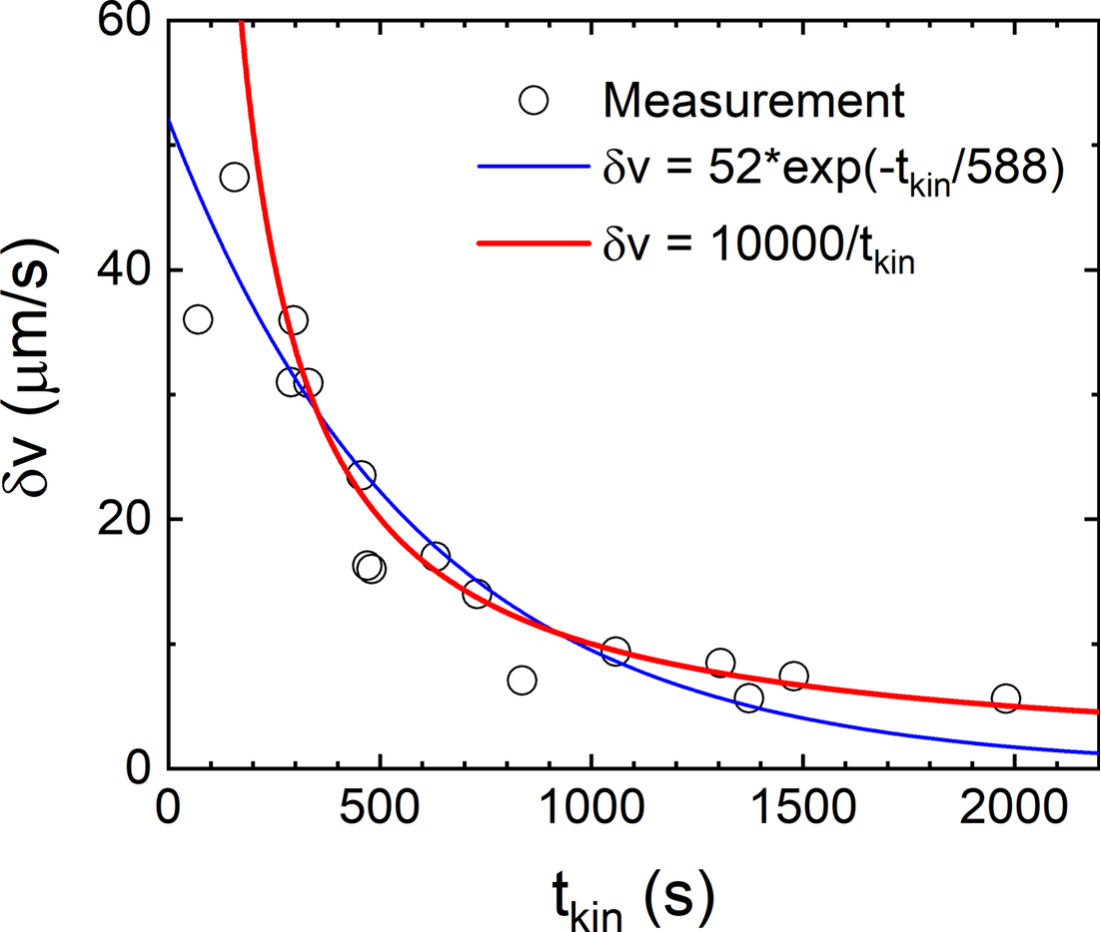
The decay of velocity fluctuations from three repetitions for the suspension consisting of silica particles (*R*_S_ ≃ 300 nm and σ_*R*_ ≃ 5.4 nm) with ϕ ≃ 0.014. The continuous lines represent the functional form given in the legend. The error bars for δ*v* are smaller than the size of the symbols.

Fig. 6 ▸ shows the typical decay of δ*v* along *q*_∥_ by combining parameters from several repetitions following the same shaking protocol. The variation along *q*_⊥_ also follows the same trend but is smaller in amplitude. It is tempting to describe this decay with an exponential function by analogy with sedimentation (Tee *et al.*, 2002 ▸; Möller & Narayanan, 2017 ▸). The corresponding function yielded an initial δ*v* ≃ 52 µm s^−1^, which is significantly smaller than what occurred during the shaking process. Alternatively, the decay can be described by a power law with exponent ∼1. Due to the large spread of the data, numerically it is difficult to distinguish which function fits better, but the asymptotic limits of an initial large value of δ*v* (*t*_kin_ > 0) and a slowly decaying tail are reproduced by a power-law decay.

**Figure 7:**
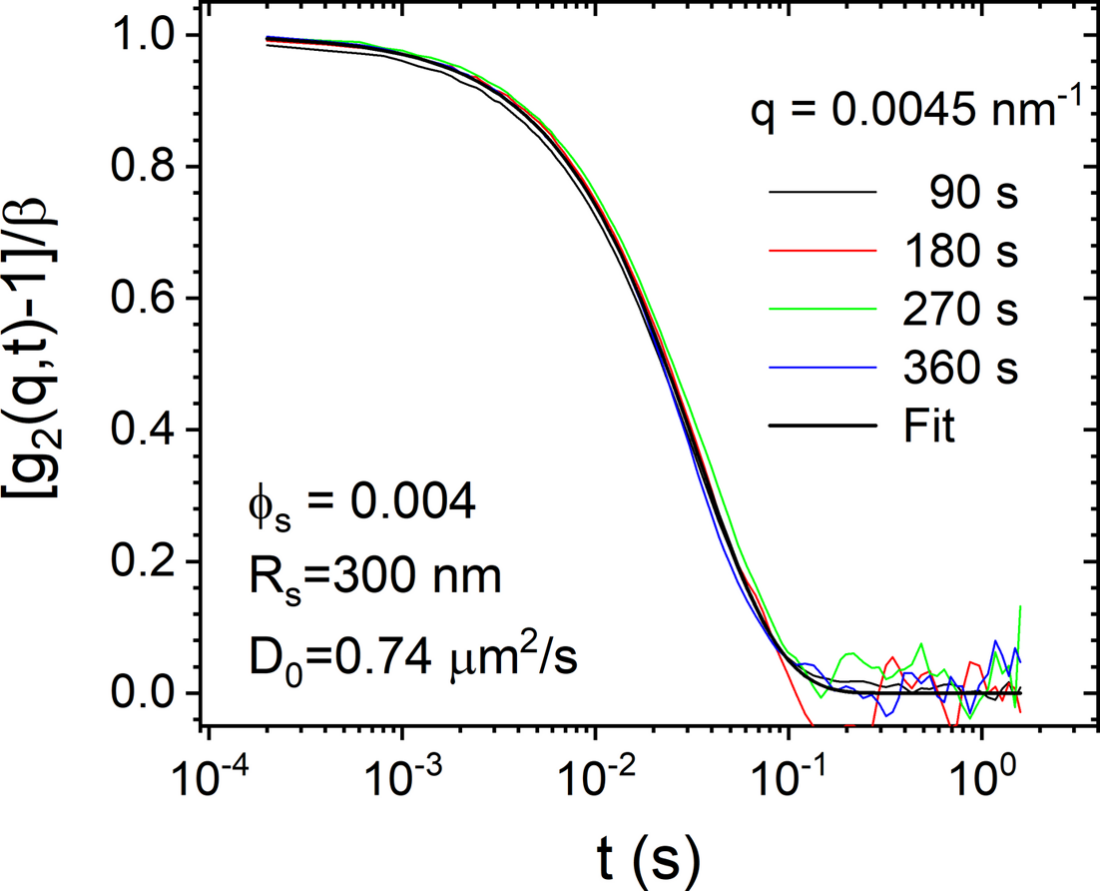
Lack of time evolution of *g*_2_(*q*, *t*) functions for a suspension of silica particles (*R*_S_ ≃ 300 nm and ϕ ≃ 0.004) at a fixed *q* of 4.5 × 10^−3^ nm^−1^. The continuous line is a fit to an exponential decay [equation (3)] with the *D*_0_ value indicated in the legend.

Surprisingly, a suspension of the same particles with a lower ϕ ≃ 0.004 did not manifest similar velocity fluctuations following the same shaking protocol. Fig. 7 ▸ presents representative *g*_2_(*q*, *t*) for this suspension at a fixed *q* = 4.5 × 10^−3^ nm^−1^. At all times, the decay of *g*_2_(*q*, *t*) is described by an exponential function with the expected value of *D*_0_ for these particles. The magnitude of δ*v* decreased with ϕ and the dynamics became indistinguishable from Brownian diffusion below ϕ ≃ 0.01. The transition region is rather fuzzy and a precise cut-off value of ϕ could not be determined from the present experiments.

In order to rationalize the observed findings, one can relate homogenization by vigorous shaking to scalar mixing in Batchelor flow (Batchelor, 1959 ▸). This is because the Reynolds number (Re) for a flow rate of 40 mm s^−1^ in a capillary of diameter 1 mm for water-like viscosity is about 40, well below the transition to fully developed turbulence. The smallest size of the local concentration fluctuations is given by the dissipation length *l*_D_ of Batchelor flow, *l*_D_ ≃ *l*Pe^−1/2^Re^−1/4^, with *l* the smallest size of the channel and the flow-generated Pe ≃ 2*R*_S_*v*/*D*_0_ (Villermaux, 2019 ▸). For the maximum *v* obtained during shaking, Pe ≃ 30000 and therefore *l*_D_ ≃ 2.3 µm. This size scale should be compared with the mean separation between particles, 〈*d*〉 = 2*R*_S_ϕ^−1/3^. For ϕ = 0.004, 〈*d*〉 ≃ 4 µm. In reality, the estimation of *v* during shaking could be off by a factor of up to 2. In that case, *l*_D_ increases to 3.8 µm. When 〈*d*〉 ≥ *l*_D_, the induced velocity fluctuations become less significant or not detectable. For smaller 〈*d*〉 (larger ϕ), the velocity fluctuations induced during the shaking process become important. δ*v* decays as Brownian diffusion homogenizes the suspension with time.

For smaller particles, 〈*d*〉 is smaller but the diffusion is faster. Therefore, these non-equilibrium velocity fluctuations may be manifested less except in the very low *q* region. Nevertheless, the same shaking protocols with a suspension of silica particles *R*_S_ ≃ 126 nm yielded similar results, as shown in Fig. S3. These velocity fluctuations can have implications in applications such as measurement of particle size and diffusion coefficients by dynamic light scattering (DLS) and XPCS when micrometre-sized particles are involved. In particular, the reproducibility may be affected by the homogenization method used prior to the measurement. In a typical DLS setup, the scattering geometry is horizontal and this effect may even go unnoticed, leading to an underestimation of *R*_H_. Finally, it is not a thermally induced effect as a temperature variation should not change the functional form of *g*_2_(*q*, *t*) (see Fig. S4) (Zinn *et al.*, 2022 ▸) and it should be direction and ϕ independent.

### 3.3. Velocity fluctuations following shear cessation

When the same colloidal suspension (*R*_S_ ≃ 300 nm and ϕ ≃ 0.014) is subjected to a uniform shear in a coaxial cylindrical cell, well defined oscillations appear in the measured *g*_2_(*q*, *t*) (Narayanan *et al.*, 2023 ▸). These oscillations are due to a constant velocity difference in the annular gap (Burghardt *et al.*, 2012 ▸; Narayanan *et al.*, 2020 ▸) and can be well described by equation (5) with α = 1 (Narayanan *et al.*, 2023 ▸). An important question is whether these velocity fluctuations persist upon cessation of uniform shear. Even at a modest shear rate (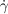 ≃ 10 s^−1^), δ*v* is large and is given by the angular frequency of the rotor and the size of the annular gap.

**Figure 8:**
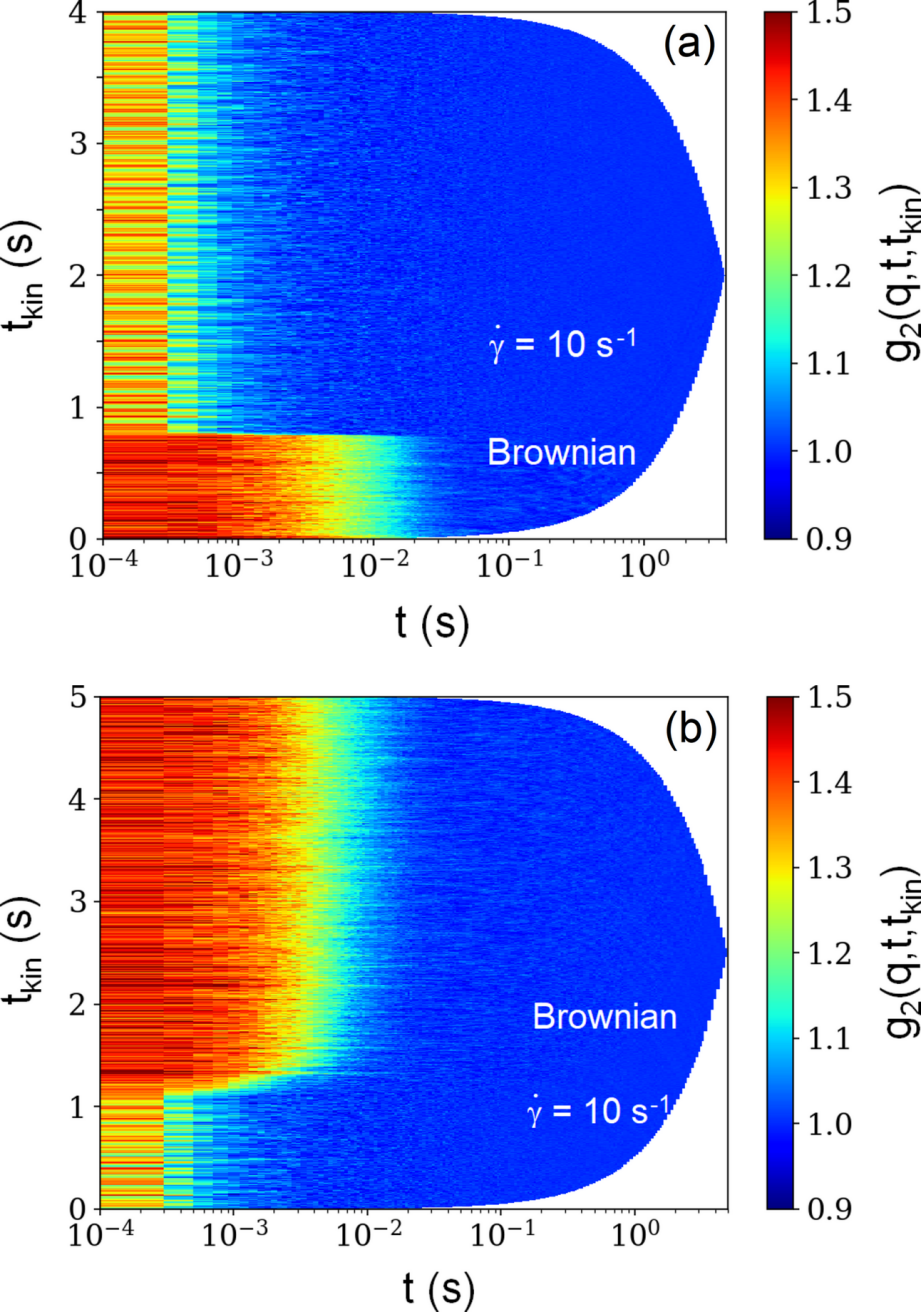
TTCF plots along the flow direction during (*a*) the startup and (*b*) the cessation of shear for a suspension of silica particles (*R*_S_ ≃ 300 nm and ϕ ≃ 0.014) at a fixed *q* of 3.4 × 10^−3^ nm^−1^. Here the origin of *t*_kin_ is the same as that of the lag time *t*. The sharp transition behaviour is identical at other *q* values.

For this experiment, the colloidal suspension was contained in the annular gap between two concentric capillaries, with the inner one coupled to the shaft of the rheometer (Narayanan *et al.*, 2020 ▸). In order to capture the fast decay due to shear (Doppler shift caused by the flow), XPCS acquisitions were performed at a frame rate of 20 kHz. The change in dynamics was monitored via TTCF (Chèvremont *et al.*, 2024 ▸). Fig. 8 ▸(*a*) illustrates the rapid change in dynamics upon switching on the shear (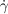 ≃ 10 s^−1^). Here the TTCF plot for a given *q* can be considered as a stack of *g*_2_(*q*, *t*) curves. Before turning on the shear, the decay is fully governed by Brownian motion and then by Doppler shift caused by advection of the particles. This change in dynamics is rapid, on the millisecond scale. Fig. 8 ▸(*b*) demonstrates the opposite scenario when the shear is turned off. Rather surprisingly, the corresponding transformation of dynamics happens within 300 ms and Brownian dynamics are fully restored on the second time scale.

This step-like transition behaviour was also observed with other values of 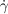, *ca* 200 s^−1^. This contrasting scenario in comparison with the shaking experiment can be rationalized on the basis of the homogeneity of shear flow. Within the coaxial flow geometry, 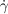 is nearly uniform across the gap and the flow does not create concentration inhomogeneities in the suspension. The hydrodynamic contributions are arrested upon cessation of shear. The inertia of the particles is negligible and Brownian dynamics are restored at once. However, in a stirred suspension the flow is inhomogeneous below the rotor and δ*v* decays on a much slower time scale (Chèvremont & Narayanan, 2025 ▸).

## 4. Summary and outlook

We have presented two representative examples of the use of UA-XPCS to probe relatively fast out-of-equilibrium dynamics in colloidal suspensions subjected to an external flow. A fourth-generation synchrotron source and an advanced photon-counting pixel array detector enabled these measurements. These examples are not exhaustive and primarily serve the purpose of illustrating the new possibilities offered by multispeckle XPCS on a faster time scale. The observed non-equilibrium fluctuations upon shaking a colloidal suspension are rather intriguing but they can play an adverse role in particle sizing in the micrometre range. A similar flow-induced non-equilibrium effect could occur when using an automated pipetting system for XPCS measurements. The static scattering profile is not affected but the diffusion coefficient from dynamic measurement may be overestimated. Therefore, it is always safer to correlate static and dynamic measurements when investigating colloidal suspensions.

In non-equilibrium thermodynamics of fluid mixtures, giant non-equilibrium fluctuations of concentration and temperature are relatively new phenomena (Sengers, 2024 ▸). There are a variety of similar non-equilibrium effects in driven colloidal suspensions which will be worth exploring by multispeckle XPCS. The intense X-ray beam available at an XFEL instrument can itself induce thermally driven out-of-equilibrium dynamics (Dallari *et al.*, 2021 ▸).

Another interesting scenario is when colloids are suspended in biological milieux (Semeraro *et al.*, 2018*a* ▸; Otto *et al.*, 2024 ▸; Silva *et al.*, 2024 ▸), where subtle non-equilibrium effects may be elicited. Out-of-equilibrium dynamics are prevalent in biology; for example, active transport in cellular media involves anomalous diffusion via Lévy flight or fractional Brownian motion (Nolte, 2024 ▸). The observed non-equilibrium dynamics of otherwise passive particles manifest certain aspects of active colloids (Zinn *et al.*, 2020 ▸; Zinn *et al.*, 2022 ▸). Therefore, such out-of-equilibrium behaviour may bridge the gap between passive and active colloids.

## Supplementary Material

Additional data. DOI: 10.1107/S1600576725001244/ju5077sup1.pdf

## References

[bb1] Abernathy, D. L., Grübel, G., Brauer, S., McNulty, I., Stephenson, G. B., Mochrie, S. G. J., Sandy, A. R., Mulders, N. & Sutton, M. (1998). *J. Synchrotron Rad.* **5**, 37–47. 10.1107/S0909049597015835 16687799

[bb2] Agarwal, B. K. (2013). *X-ray spectroscopy: an introduction.* Heidelberg: Springer.

[bb3] Batchelor, G. K. (1959). *J. Fluid Mech.* **5**, 113–133.

[bb4] Batchelor, G. K. (1972). *J. Fluid Mech.* **52**, 245–268.

[bb5] Burghardt, W. R., Sikorski, M., Sandy, A. R. & Narayanan, S. (2012). *Phys. Rev. E*, **85**, 021402. 10.1103/PhysRevE.85.021402 22463207

[bb6] Busch, S., Jensen, T. H., Chushkin, Y. & Fluerasu, A. (2008). *Eur. Phys. J. E*, **26**, 55. 10.1140/epje/i2007-10305-2 18415042

[bb7] Caflisch, R. E. & Luke, J. H. (1985). *Phys. Fluids*, **28**, 759–760.

[bb8] Chèvremont, W. & Narayanan, T. (2025). *Phys. Fluids*, **37**, 023390.

[bb9] Chèvremont, W., Zinn, T. & Narayanan, T. (2024). *J. Synchrotron Rad.* **31**, 65–76. 10.1107/S1600577523008627 PMC10833426 37933847

[bb10] Dallari, F., Jain, A., Sikorski, M., Möller, J., Bean, R., Boesenberg, U., Frenzel, L., Goy, C., Hallmann, J., Kim, Y., Lokteva, I., Markmann, V., Mills, G., Rodriguez-Fernandez, A., Roseker, W., Scholz, M., Shayduk, R., Vagovic, P., Walther, M., Westermeier, F., Madsen, A., Mancuso, A. P., Grübel, G. & Lehmkühler, F. (2021). *IUCrJ*, **8**, 775–783. 10.1107/S2052252521006333 PMC8420773 34584738

[bb11] Eriksson, M., van der Veen, J. F. & Quitmann, C. (2014). *J. Synchrotron Rad.* **21**, 837–842. 10.1107/S1600577514019286 25177975

[bb12] Fröjdh, E., Bergamaschi, A. & Schmitt, B. (2024). *Front. Phys.* **12**, 1304896.

[bb13] Glatter, O. (2002). *Neutrons, X-rays and light: scattering methods applied to soft condensed matter*, pp. 73–102. Amsterdam: Elsevier Science.

[bb14] Goodman, J. W. (1985). *Statistical optics.* New York: John Wiley & Sons.

[bb15] Grübel, G. & Zontone, F. (2004). *J. Alloys Compd.* **362**, 3–11.

[bb16] Guazzelli, É. & Hinch, J. (2011). *Annu. Rev. Fluid Mech.* **43**, 97–116.

[bb17] Hirano, M. & Norisuye, T. (2024). *Phys. Fluids*, **36**, 113370.

[bb18] Lehmkühler, F., Roseker, W. & Grübel, G. (2021). *Appl. Sci.* **11**, 6179.

[bb19] Möller, J. & Narayanan, T. (2017). *Phys. Rev. Lett.* **118**, 198001. 10.1103/PhysRevLett.118.198001 28548515

[bb20] Narayanan, T. (2024). *Adv. Colloid Interface Sci.* **325**, 103114. 10.1016/j.cis.2024.103114 38452431

[bb21] Narayanan, T., Chèvremont, W. & Zinn, T. (2023). *J. Appl. Cryst.* **56**, 939–946. 10.1107/S1600576723004971 PMC10405582 37555224

[bb22] Narayanan, T., Dattani, R., Möller, J. & Kwaśniewski, P. (2020). *Rev. Sci. Instrum.* **91**, 085102. 10.1063/5.0012905 32872916

[bb23] Narayanan, T., Sztucki, M., Zinn, T., Kieffer, J., Homs-Puron, A., Gorini, J., Van Vaerenbergh, P. & Boesecke, P. (2022). *J. Appl. Cryst.* **55**, 98–111. 10.1107/S1600576721012693 PMC8805168 35145357

[bb24] Nolte, D. D. (2024). *Rep. Prog. Phys.* **87**, 036601.

[bb25] Otto, F., Dallari, F., Westermeier, F., Wieland, D. F., Parak, W. J., Lehmkühler, F. & Schulz, F. (2024). *Aggregate*, **5**, e483.

[bb26] Padding, J. T. & Louis, A. A. (2008). *Phys. Rev. E*, **77**, 011402. 10.1103/PhysRevE.77.011402 18351852

[bb27] Paleo, P., Kieffer, J. & Chushkin, Y. (2021). *Dynamix v0.2: XPCS from Python*, https://doi.org/10.5281/zenodo.5520626.

[bb28] Pavlinsky, G. V. (2021). *X-ray Spectrom.* **50**, 454–457.

[bb29] Petukhov, A. V., Meijer, J.-M. & Vroege, G. J. (2015). *Curr. Opin. Colloid Interface Sci.* **20**, 272–281.

[bb30] Pusey, P. (2002). *Neutrons, X-rays and light: scattering methods applied to soft condensed matter*, pp. 3–21. Amsterdam: Elsevier Science.

[bb31] Raimondi, P., Carmignani, N., Carver, L., Chavanne, J., Farvacque, L., Le Bec, G., Martin, D., Liuzzo, S., Perron, T. & White, S. (2021). *Phys. Rev. Accel. Beams*, **24**, 110701.

[bb32] Ramaswamy, S. (2001). *Adv. Phys.* **50**, 297–341.

[bb33] Segrè, P., Herbolzheimer, E. & Chaikin, P. (1997). *Phys. Rev. Lett.* **79**, 2574–2577.

[bb34] Segrè, P. N. (2016). *Fluids, colloids and soft materials: an introduction to soft matter physics*, edited by A. Fernandez-Nieves & A. M. Puertas, ch.4, pp. 45–58. Hoboken: John Wiley & Sons.

[bb35] Semeraro, E. F., Devos, J. M. & Narayanan, T. (2018*a*). *J. Chem. Phys.* **148**, 204905. 10.1063/1.5026778 29865804

[bb36] Semeraro, E. F., Möller, J. & Narayanan, T. (2018*b*). *J. Appl. Cryst.* **51**, 706–713.

[bb37] Sengers, J. V. (2024). *Int. J. Thermophys.* **45**, 132.

[bb38] Shin, S. (2021). *AAPPS Bull.* **31**, 21.

[bb39] Silva, C. E., Picco, A. S., Galdino, F. E., de Burgos Martins de Azevedo, M., Cathcarth, M., Passos, A. R. & Cardoso, M. B. (2024). *Nano Lett.* **24**, 13293–13299. 10.1021/acs.nanolett.4c03662 PMC11505373 39361530

[bb40] Sutton, M. (2008). *C. R. Phys.* **9**, 657–667.

[bb41] Sztucki, M. (2021). *SAXSutilities*, https://www.saxsutilities.eu.

[bb42] Tee, S.-Y., Mucha, P., Cipelletti, L., Manley, S., Brenner, M., Segre, P. & Weitz, D. (2002). *Phys. Rev. Lett.* **89**, 054501. 10.1103/PhysRevLett.89.054501 12144444

[bb43] Veen, F. van der & Pfeiffer, F. (2004). *J. Phys. Condens. Matter*, **16**, 5003–5030.

[bb44] Villermaux, E. (2019). *Annu. Rev. Fluid Mech.* **51**, 245–273.

[bb45] Zhang, Q., Dufresne, E. M., Narayanan, S., Maj, P., Koziol, A., Szczygiel, R., Grybos, P., Sutton, M. & Sandy, A. R. (2018). *J. Synchrotron Rad.* **25**, 1408–1416. 10.1107/S1600577518009074 30179180

[bb46] Zinn, T., Homs, A., Sharpnack, L., Tinti, G., Fröjdh, E., Douissard, P.-A., Kocsis, M., Möller, J., Chushkin, Y. & Narayanan, T. (2018). *J. Synchrotron Rad.* **25**, 1753–1759. 10.1107/S1600577518013899 PMC6225738 30407186

[bb47] Zinn, T., Narayanan, T., Kottapalli, S. N., Sachs, J., Sottmann, T. & Fischer, P. (2022). *New J. Phys.* **24**, 093007.

[bb48] Zinn, T., Sharpnack, L. & Narayanan, T. (2020). *Phys. Rev. Res.* **2**, 033177.

