# Supplementary material for: Probing the out-of-equilibrium dynamics of driven colloids by X-ray photon correlation spectroscopy
Source: J Appl Crystallogr. 2025 Mar 7;58(Pt 2):535–42. doi: 10.1107/S1600576725001244 (PMC11957404; doi:10.1107/S1600576725001244)
Supplement: Supplementary file 1 [file j-58-00535-sup1.pdf]

JOURNAL OF
APPLIED
CRYSTALLOGRAPHY

Volume 58 (2025)

Supporting information for article:

Probing the out-of-equilibrium dynamics of driven colloids by X-ray photon correlation spectroscopy

Theyencheri Narayanan, William Chèvremont and Thomas Zinn

Supporting Information: Probing the Out-of-Equilibrium Dynamics of Driven Colloids by XPCS

Theyencheri Narayanan,[a]* William Chèvremont[a] and Thomas Zinn[a,b]

[a]ESRF - The European Synchrotron, 38043 Grenoble, France, and [b]Present address: Diamond Light Source, Didcot OX11 0DE, United Kingdom.

E-mail: narayan@esrf.fr

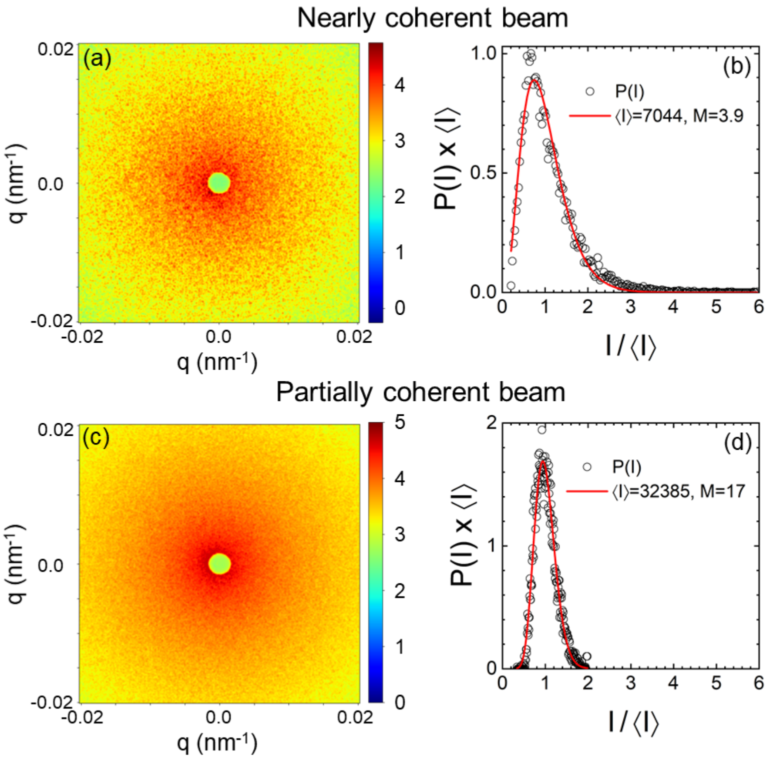

Fig. 1. Static speckle patterns from an alumina powder sample measured with two different collimation settings. The intensity statistics, $P(I)$, were analyzed over the range $2.0 \times 10^{-3}\,\mathrm{nm}^{-1} \leq q \leq 3.2 \times 10^{-3}\,\mathrm{nm}^{-1}$ that covered the most intense part of the pattern. (a) Slits closed to $40\,\mu$m vertically and $20\,\mu$m horizontally. (b) Corresponding analysis of $P(I)$, using Equation (1) in the main text. (c) The speckle pattern of the same sample recorded with a larger beam, slits opened to $100\,\mu$m x $100\,\mu$m. (d) Corresponding $P(I)$ and analysis using Equation (1).

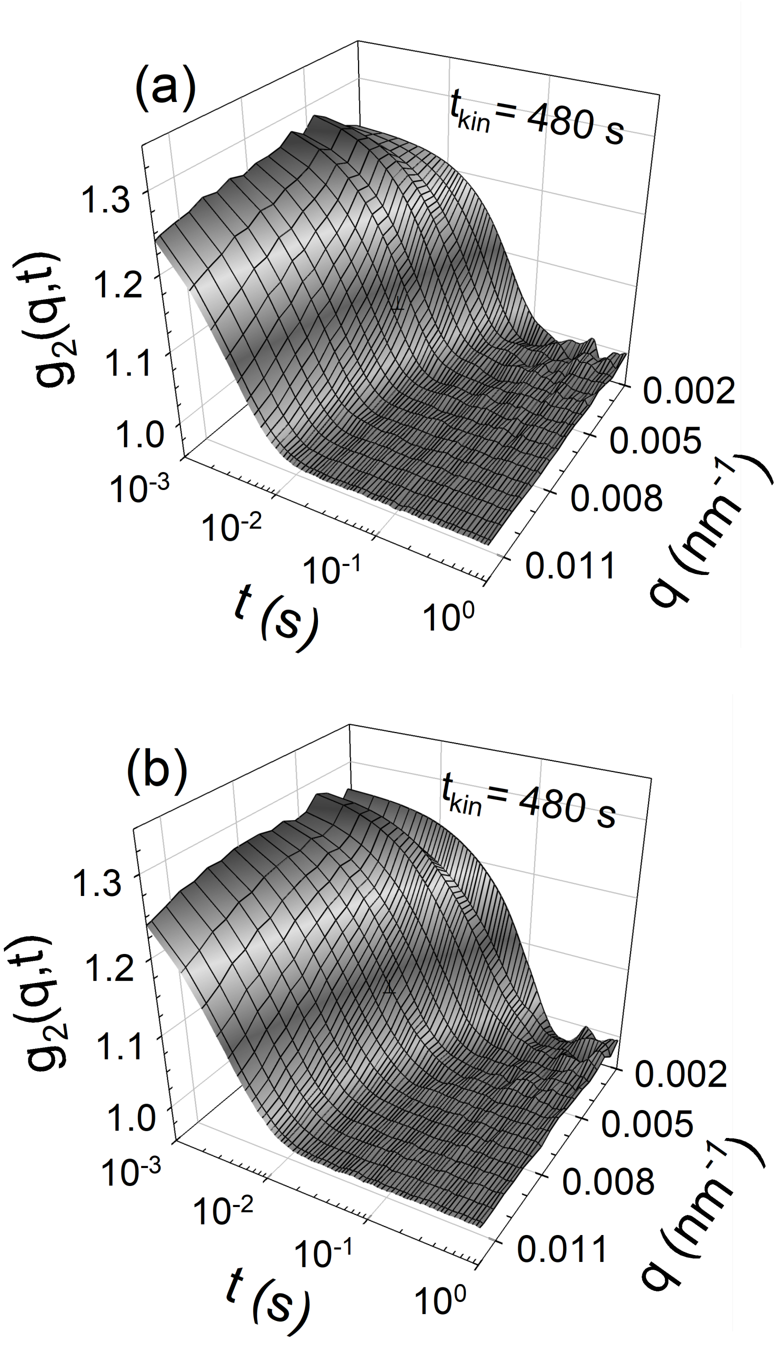

Fig. 2. Comparison of $g_2(q,t)$ along the two directions for a suspension of silica particles ($R_S \simeq 300\,\mathrm{nm}$ and $\sigma_R \simeq 5.4\,\mathrm{nm}$) with $\phi \simeq 0.014$ at 480 s after the shaking. Notice (a) relatively faster decay along $q_{\|}$ and (b) slower decay along $q_{\perp}$.

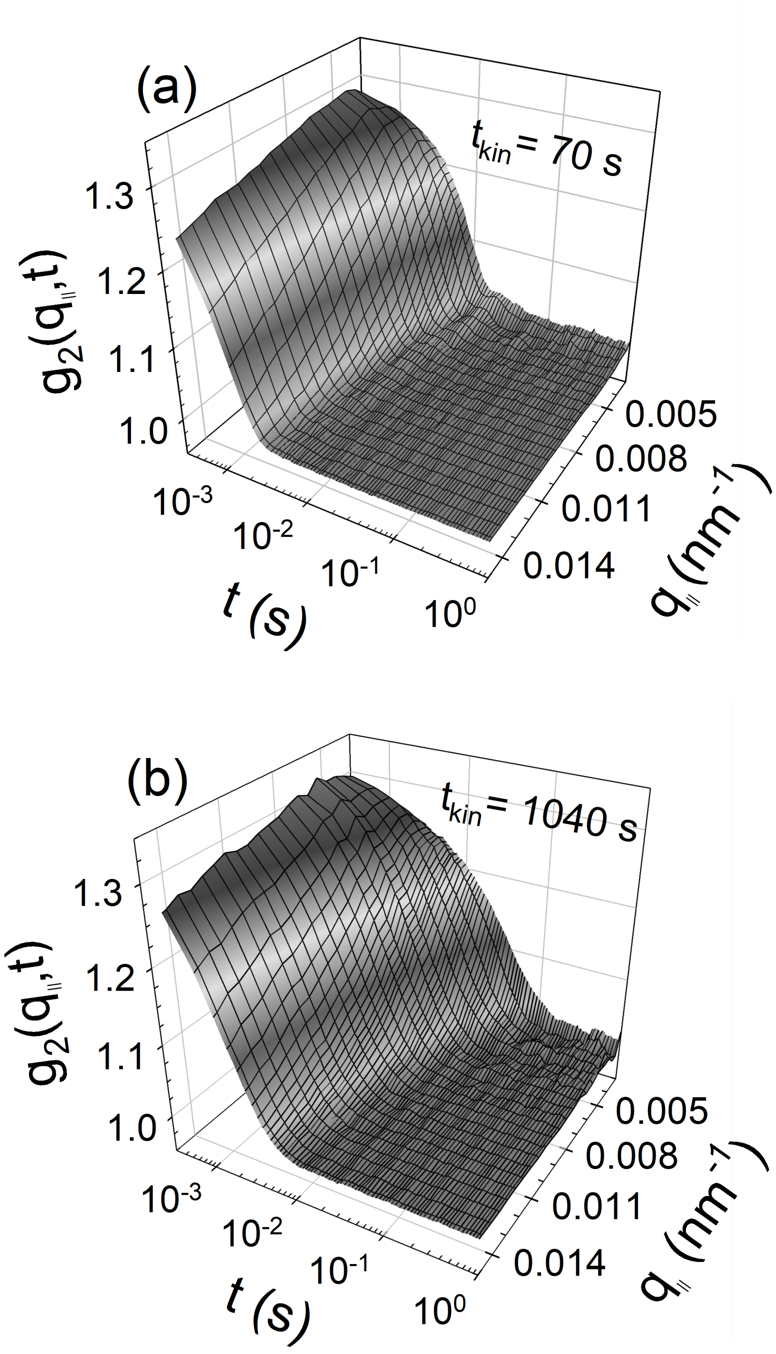

Fig. 3. The time evolution of $g_2(q,t)$ along $q_{\|}$ for a suspension of silica particles ($R_S \simeq$ 126 nm and $\sigma_R \simeq 6.0$ nm) with $\phi \simeq 0.1$. (a) 70 s after the shaking illustrating the signature of velocity fluctuations and (b) at about 1000 s while the suspension is gradually returning to Brownian dynamics.

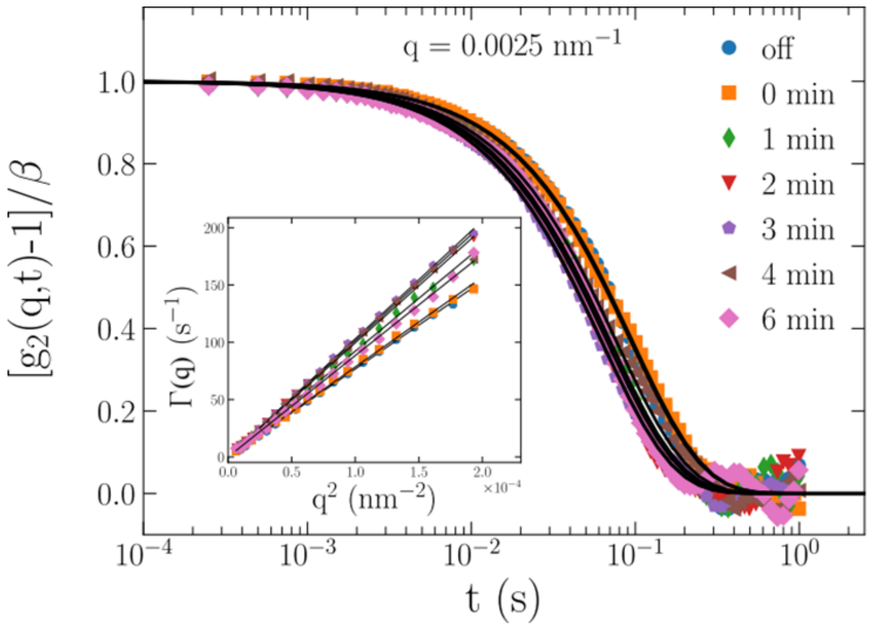

Fig. 4. Speeding up of the dynamics upon heating an aqueous colloidal suspension ($R_S \simeq 300\,\mathrm{nm}$ and $\sigma_R \simeq 5.4\,\mathrm{nm}$ with $\phi \simeq 0.01$) from 22 °C to 32 °C by UV illumination. The functional form of $g_2(q,t)$ remains exponential and only the relaxation rate $\Gamma(q)$ increases due to the reduction of viscosity. The inset demonstrates the expected diffusive dynamics, $\Gamma(q) = D_0 q^2$.
